# World-Wide Prevalence and Genotype Distribution of Enteroviruses

**DOI:** 10.3390/v13030434

**Published:** 2021-03-08

**Authors:** Lieke Brouwer, Giulia Moreni, Katja C. Wolthers, Dasja Pajkrt

**Affiliations:** 1 Department of Medical Microbiology, Amsterdam UMC, Meibergdreef 9, 1105 AZ Amsterdam, The Netherlands; g.moreni@amsterdamumc.nl (G.M.); k.c.wolthers@amsterdamumc.nl (K.C.W.); 2 Department of Pediatric Infectious Diseases, Amsterdam UMC, Meibergdreef 9, 1105 AZ Amsterdam, The Netherlands; d.pajkrt@amsterdamumc.nl

**Keywords:** enterovirus, epidemiology, prevalence, genotype

## Abstract

Enteroviruses (EVs) are highly prevalent viruses world-wide, causing a wide range of diseases in both children and adults. Insight in the global prevalence of EVs is important to define their clinical significance and total disease burden, and assists in making therapeutic decisions. While many studies have been conducted to describe epidemiology of EVs in specific (sub)populations and patient cohorts, little effort has been made to aggregate the available evidence. In the current study, we conducted a search in the PubMed and Embase (Ovid) databases to identify articles reporting EV prevalence and type distribution. We summarized the findings of 153 included studies. We found that EVs are highly prevalent viruses in all continents. *Enterovirus B* was the most detected species worldwide, while the other species showed continent-specific differences, with *Enterovirus C* more detected in Africa and *Enterovirus A* more detected in Asia. Echovirus 30 was by far the most detected type, especially in studies conducted in Europe. EV types in species *Enterovirus B*—including echovirus 30—were often detected in patient groups with neurological infections and in cerebrospinal fluid, while *Enterovirus C* types were often found in stool samples.

## 1. Introduction

Enteroviruses (EVs) are among the most prevalent viruses infecting humans worldwide. There are 106 types currently known to infect humans, of which dozens are frequently detected globally [1,2,3,4]. Most of these types were defined before sequencing techniques were available on a large scale, and typing was performed based on the biological properties of these viruses in cell culture or mouse models. The EV types that were identified during that period of time are still called coxsackie A viruses (CVA), coxsackie B viruses (CVB), echoviruses (E) and polioviruses (PV). The newer types have been defined by analysis of their viral protein 1 (VP1) region sequence, and their names consist of ‘Enterovirus’, their species letter, and a consecutive number, starting with Enterovirus D68 (EV-D68) [5]. Each EV type that has been identified in humans falls into one of four species; *Enterovirus A, B, C* or *D*. These species vary in size; species *Enterovirus B* contains more than half of all known EVs (*n* = 59), while only 4 *Enterovirus D* types have been defined (Table 1).

EVs are known to cause a wide spectrum of diseases, including encephalitis, meningitis, myocarditis, hand-foot-mouth disease (HFMD), conjunctivitis, respiratory disease and gastro-intestinal disease, but the majority of infections remain asymptomatic [6,7,8,9,10,11,12]. Some of these clinical symptoms are linked to specific types, such as EV-A71 and CVA16 to HFMD and CVB3 to myocarditis—while the clinical relevance for many of the types known to infect humans, especially for those discovered in the past 20 years, is not well-defined. Many prevalence studies have been conducted to describe EV prevalence and the distribution of the distinct EV types in cohorts of patients with specific symptoms—such as gastro-enteritis, influenza-like illness or meningitis—and in asymptomatic cohorts. In addition, surveillance studies report EV prevalence and EV types detected in larger populations with less well-defined symptoms, such as all individuals tested for EVs at a national level [2,4].

Over the years, prevalence and surveillance studies have made use of different methods to detect and type EVs. Whereas cell culture has been applied for many years to detect presence of EVs in clinical samples, this changed with the introduction of sequencing. The currently recommended method of detection is by a PCR targeting the 5’ untranslated region (UTR) of the virus, which has proven to be more sensitive than culture [13,14,15]. The transition from serotyping by neutralization assays to genotyping by sequencing the VP1 region has also increased sensitivity; as not all types could be isolated in the cell lines used, and antibodies are lacking for strains detected after EV-A71, many types could not be serotyped [16].

Though many studies on EV prevalence have been conducted in either specific (sub)populations or patient cohorts, few efforts have been made to aggregate the epidemiological evidence to clearly display the worldwide prevalence, related type-distribution of EVs and their clinical relevance. A review by Janes et al. including several large surveillance studies—mainly applying virus isolation and serotyping methods—showed that globally, *Enterovirus B* was the most prevalent species, with E30 and several other *Enterovirus B* types as the most detected types. *Enterovirus A* appeared to be the predominant species in Asia, mainly resulting from large outbreaks of HFMD caused by CVA16 and EVA71 [3]. The aim of this current review was to describe worldwide EV prevalence and the clinical relevance of different EV species and types by their distribution in different continents and clinical cohorts.

## 2. Methods

### 2.1. Literature Search and Data Extraction

On 12 June 2020, we performed a literature search in the PubMed and Embase (Ovid) databases (Table 2), to identify publications on EV prevalence and type distribution. After removal of duplicates, articles were screened on title and abstract and subsequently on full text based on the inclusion and exclusion criteria by two reviewers (Table 2). As the recommended methods to detect and type EVs are by PCR targeting the 5’UTR and sequencing the VP1 region, respectively, only studies that applied these methods were included. Studies that described EV prevalence and/or type distribution in a clinical cohort with EV-type specific symptoms (e.g., HFMD or myocarditis) were excluded, as well as studies on outbreaks of EVs, as they would wrongfully inflate prevalence numbers and skew the type distribution towards specific types.

Articles were included for analysis on EV prevalence or analysis on EV type distribution, or both. We extracted data on the number of samples tested and typed, continent and country where the study was conducted, symptoms of the included participants, age of the participants, sample type, EV prevalence and EV types detected from the included articles. When no data on number of samples was available, number of included patients was taken as a proxy. Sample type was recorded as one of five categories; gastrointestinal (fecal samples and rectal swabs), cerebrospinal fluid (CSF), respiratory (e.g., nasopharyngeal swabs and bronchoalveolar lavage), blood (including serum and plasma) and multiple sample types. Clinical complaints were recorded as one of five categories; gastrointestinal, neurological, respiratory, other or no symptoms. When the age limit of a pediatric population was not specified, it was assumed individuals up to 18 years of age were included. The quality of all included articles was assessed using quality control checklists for cross sectional and case–control studies (Tables S1 and S2).

### 2.2. Statistical Analysis

Median EV prevalence and interquartile ranges (IQRs) weighted for the number of tested samples were calculated overall and for different continents, symptom groups, age groups and sample types. The proportion of each EV species was calculated for continents for which at least ten studies describing EV type distribution were available. Proportions corrected for the number of types per species (Table 1) were calculated as well. The distribution of EV types was determined both globally, and in different continents, symptom groups and sample types. All analyses were performed using R version 3.5.3.

## 3. Results

### 3.1. The Included Studies

**Figure 1:**
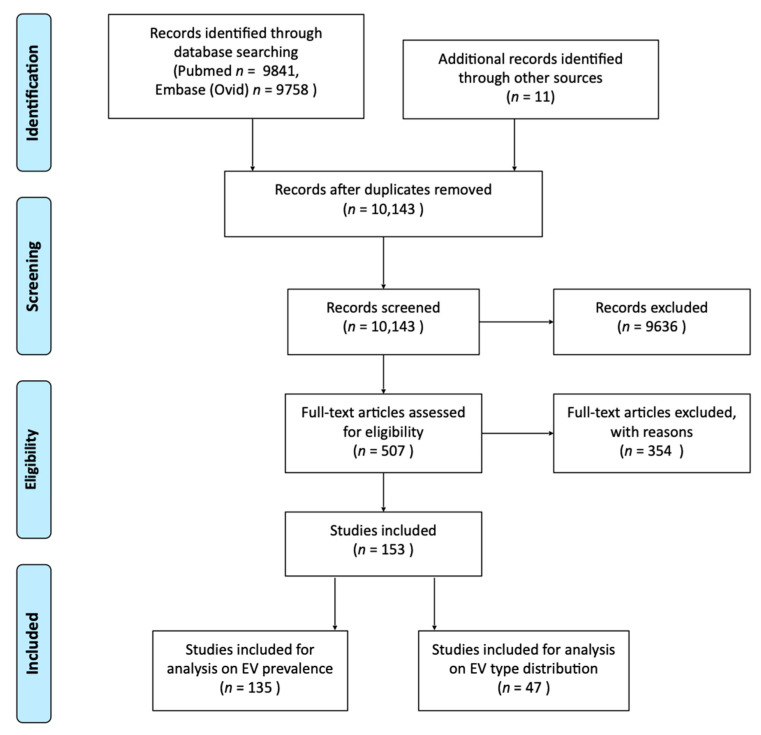
Flow diagram displaying the number of studies identified, screened assessed for eligibility and included [17].

A total of 10,132 unique studies were identified in the search in the PubMed and Embase (Ovid) databases. Eleven papers were added during the in- and exclusion through chain-referral sampling. Screening on title and abstract and full text resulted in exclusion of 9636 and 354 articles, respectively. A total of 153 studies were included for the current study, of which 135 were included for analysis on EV prevalence and 47 were included for analysis on EV type distribution (Figure 1, Table S3). The quality of the studies ranged between 2/7 and 7/7 (Table 3).

**Figure 2:**
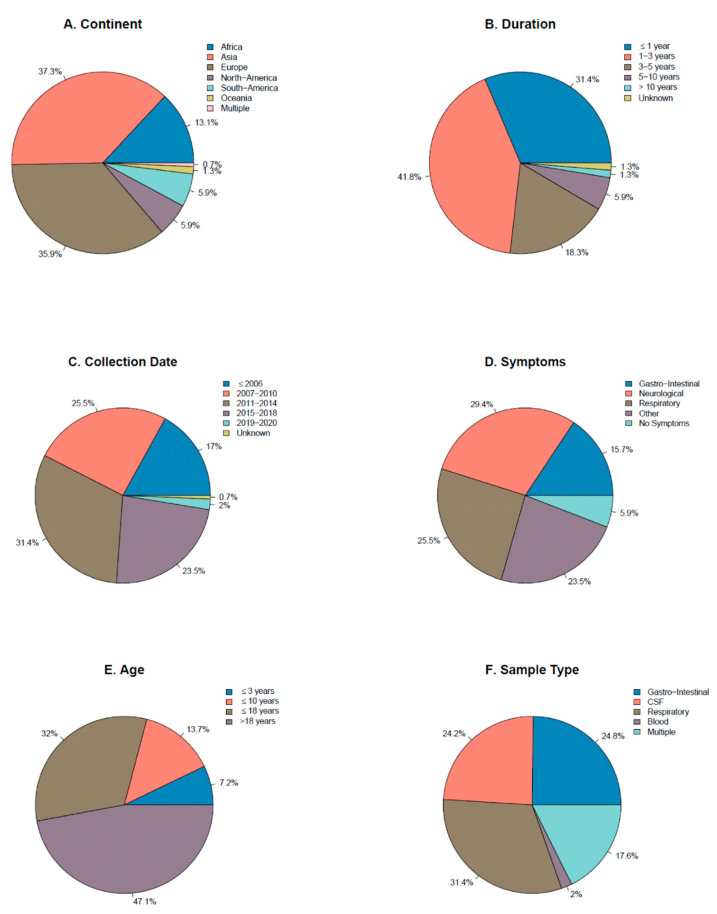
Pie charts showing the characteristics of the included studies; (**A**) Continent where the study was conducted (**B**) Duration of the inclusion period (**C**) Year in which the final inclusion/sample collection took place (**D**) The sample type collected (**E**) The symptoms of the included study participants (**F**) The age of the included participants. The ‘>18’ category includes studies in both children and adults and in exclusively adults. CSF; cerebrospinal fluid.

Of the total number of studies, most were conducted in Europe (35.9%) and Asia (37.3%), followed by Africa (13.1%) and North and South America (both 5.9%) (Figure 2A). The duration of the inclusion period in 31.4% of the studies was one year or less, while 41.8% had an inclusion period of one to three years. Only two studies (1.3%) had an inclusion period longer than ten years (Figure 2B). Only 17.0% of the studies had their last inclusion before 2006, while 26.5%, 31.4% and 23.5% had their last inclusion date before 2010, 2014 and 2018, respectively (Figure 2C). A large proportion of studies focused on patient cohorts with neurological symptoms (including sepsis-like symptoms) (29.4%) or respiratory symptoms (including influenza-like illness) (25.5%). Studies including patients with other symptoms or a variety of symptoms (often described as ’suspected EV infection’) and studies that included samples sent in for diagnostics were grouped together as ’other’ (23.5%) (Figure 2D). Most studies included exclusively children; 7.2% of the studies included children up to three years, 13.7% up to 10 years and 32.0% up to 18 years. Less than half (47.1%) of the studies included both children and adults, or exclusively adults (Figure 2E). Gastro-intestinal samples (24.8%), CSF (24.2%%) and respiratory samples (31.4%) were the sample types most often tested, while 17.6% of the studies tested multiple sample types, in some cases also including urine or vesicular fluid from blisters (Figure 2F).

### 3.2. Worldwide EV Prevalence and Type Distribution

**Figure 3:**
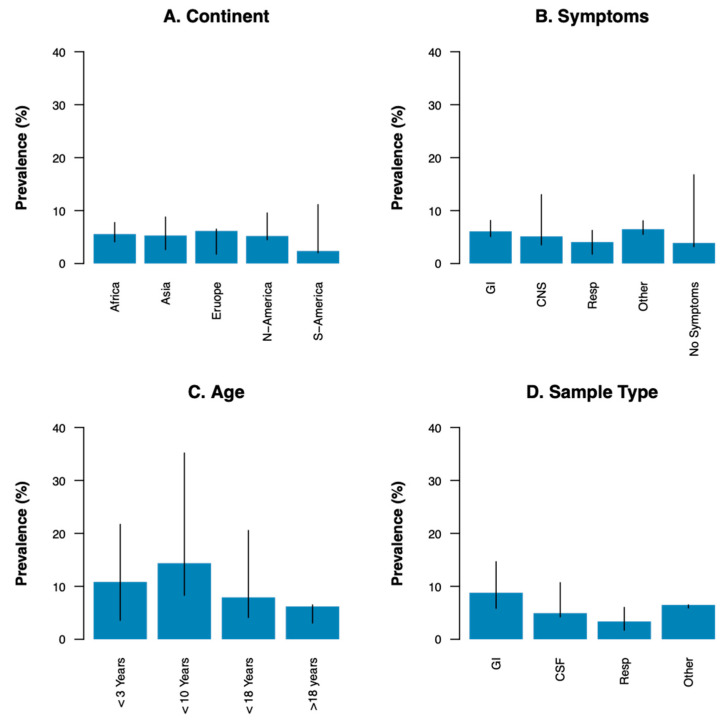
Weighted median prevalence of EVs by (**A**) Continent, (**B**) Sample type, (**C**) Symptoms of the participants and (**D**) Age of the participants. N-America; North America, S-America; South America, GI; gastrointestinal, Resp; respiratory, CNS; central nervous system, CSF; cerebrospinal fluid.

Of the 153 included studies, 135 reported on EV prevalence. Overall, the range in prevalence reported in the studies was wide, ranging from 0% in a Portuguese study on respiratory samples obtained from elderly with acute respiratory infections [18] to 89.9% in a Malawian cohort consisting of children under 5 years of age with and without anemia [10]. The median prevalence weighted for the number of samples tested in each study was 6.3% (IQR 3.2–8.1%). The median EV prevalence in different continents ranged from 2.4% (in South America) to 6.2% (in Europe) (Figure 3A).

Most included studies were conducted on the Northern hemisphere, and reported peaks in incidence mainly in summer and fall (June–November) [19,20,21,22,23,24,25,26,27,28,29,30,31,32,33,34,35]. One study from Brazil—one of the few studies performed on the Southern Hemisphere—reported that EV prevalence was higher in summer (December–March) [36].

**Figure 4:**
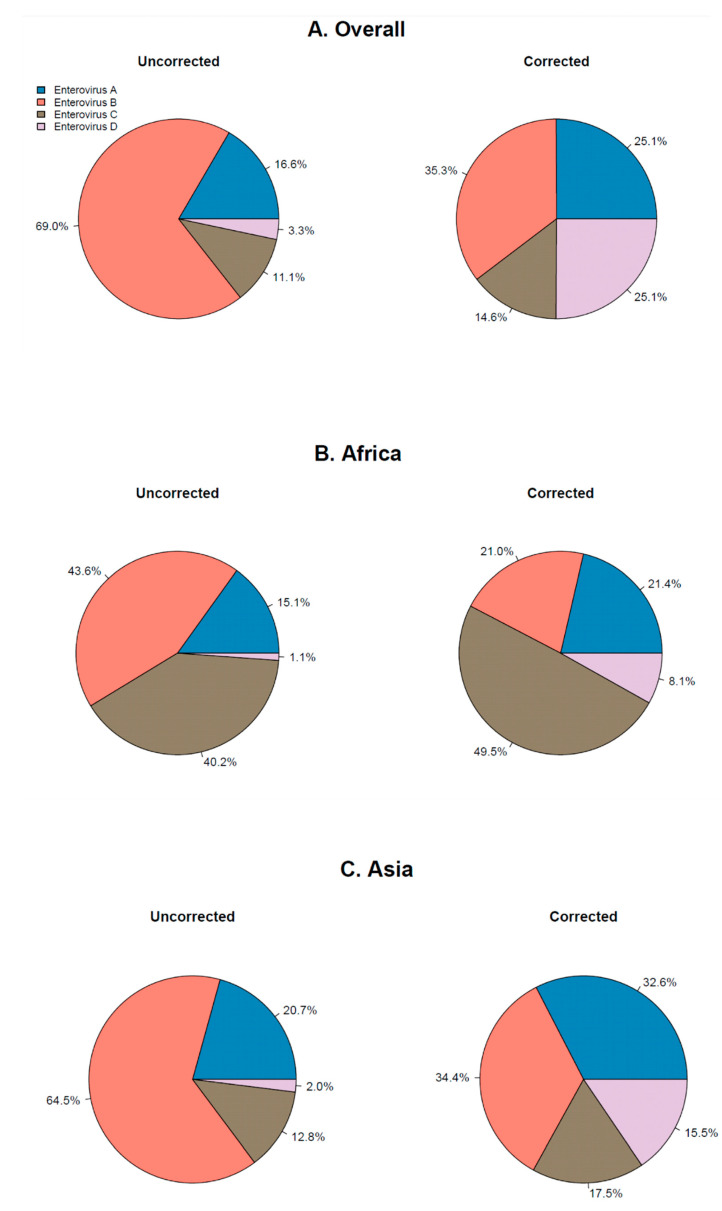

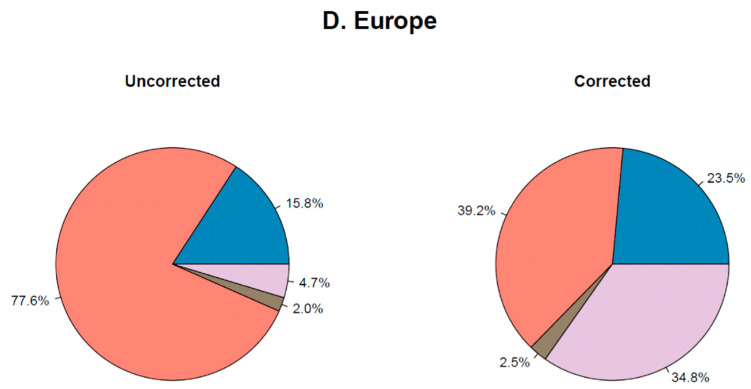
Pie charts displaying the species distribution reported in (**A**) all studies describing EV type distribution and in studies conducted in (**B**) Africa, (**C**) Asia and (**D**) Europe. Uncorrected pies display the distribution as reported by the studies. Corrected pie charts display the distribution corrected for the number of types in the species (20 in *Enterovirus A*, 59 in *Enterovirus B*, 23 in *Enterovirus C* and 4 in *Enterovirus D*).

Of the 153 included studies, 47 were included for analysis on EV type distribution. A total of 82 EV types were detected in these studies, among which 17 types in species *Enterovirus A*, 47 types in species *Enterovirus B*, 17 types in species *Enterovirus C* and one type in species *Enterovirus D*. Species distribution was calculated overall, and for Africa, Asia and Europe (Figure 4). North and South America and Oceania were excluded from analysis on continent-specific species distribution, as only nine, nine and one studies describing EV type distribution were included for these continents, respectively. Of the four species, *Enterovirus B* was the most prevalent overall (Figure 4A). Corrected for the number of types per species (20 for *Enterovirus A*, 59 for *Enterovirus B*, 23 for *Enterovirus C* and 4 for *Enterovirus D*) (Table 1), *Enterovirus B* remained the predominant species in both Asia and Europe (Figure 4C,D) and *Enterovirus C* was the predominant species in Africa (Figure 4B). The proportion of *Enterovirus A* was especially high in Asia, while *Enterovirus D* was the second largest species in Europe (Figure 4C,D).

**Figure 5:**
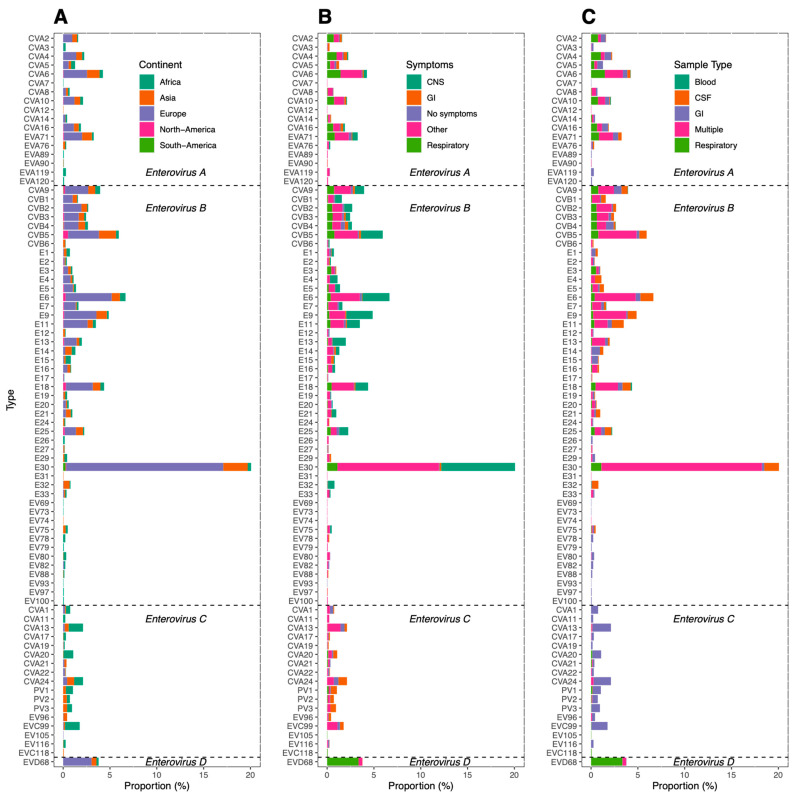
EV type distribution reported by all studies describing EV type distribution. The proportion of each reported type among the total number of reported typed strains is shown, colored by (**A**) continent, (**B**) symptoms and (**C**) sample types. GI; gastrointestinal, CNS; central nervous system, CSF; cerebrospinal fluid.

Figure 5A shows the prevalence of individual virus types. Types CVA9, CVB1-5 and several echoviruses—all within species *Enterovirus B*—were especially prevalent, with echovirus 30 (E30) ranking highest. CVA6, CVA16 and EV-A71 were the most prevalent types in species *Enterovirus A*. In species *Enterovirus C*, CVA13, CVA24 and EV-C99 were the three types with highest prevalence. Species *Enterovirus D* was represented only by type EV-D68. E30 was prevalent in Europe, while it was rarely found in Africa. In contrast, *Enterovirus C* types CVA13, CVA20 and EV-C99, rarely found in Asia and Europe, were prevalent in Africa (Figure 5A). The studies that described EV type distribution in North America identified only types within *Enterovirus B* and CVA6, CVA10, CVA16 and EV-A71 within *Enterovirus A*. CVA9, CVB4 and E30 were the only types identified in the single included study describing type distribution conducted in South-America.

### 3.3. EV Symptomatology

Median prevalence calculated from studies including patient cohorts with gastro-intestinal, neurological, respiratory or other symptoms ranged between 3.9% and 6.5% (Figure 3B). The group ‘other’ consisted mainly of studies on cohorts of patients with clinically suspected (EV) infection. In patients with neurological symptoms, types within species *Enterovirus B* were especially predominant. Studies on cohorts of patients with respiratory symptoms often reported CVA types in *Enterovirus A*, *Enterovirus D* type EV-D68 and CVB1-5 in *Enterovirus B* (Figure 5B).

Median EV prevalence was higher in studies exclusively including children up to ten years of age (14.4%) compared to studies including children up to three (10.8%) or eighteen years of age (7.9%). The median prevalence in studies including adults was lowest at 6.2% (Figure 3C). EV prevalence reported in studies with adult participants (>18 years) only was even lower, ranging from 0% to 3.8% [18,37]. Several studies reported a higher prevalence in (young) children than in adults [19,26,29,30,35,38,39,40,41,42,43]. Some studies saw a higher prevalence in young infants under three months of age compared to older children [44,45,46,47,48].

The median prevalence in gastro-intestinal samples was 8.8%, while the median prevalence in CSF and respiratory samples was 4.9% and 3.4%, respectively. The median prevalence in studies that included multiple sample types was 6.5% (Figure 3D). Most types in both species *Enterovirus A* and *Enterovirus B* were detected in a variety of sample types, often including both respiratory and gastrointestinal samples. Types in *Enterovirus C* were detected almost exclusively in gastrointestinal samples, while *Enterovirus D* type EV-D68 was detected mainly in respiratory samples (Figure 5C). Studies including patients with neurological symptoms and studies on CSF samples showed high proportions of CVBs and echovirus types in *Enterovirus B*. Studies including patients with respiratory symptoms and studies on respiratory samples mainly detected CVAs and CVBs in *Enterovirus A* and *Enterovirus B*, and EV-D68 in *Enterovirus D* (Figure 5B,C).

## 4. Discussion

In the current review, we presented an overview of worldwide EV prevalence and type distribution. We found that EVs are highly prevalent globally, with comparable EV prevalence rates across continents. *Enterovirus B* was the most detected species worldwide. However, it is also the species that contains more than half of the known EV types. After correction for the number of types per species, *Enterovirus B* was still the most detected species. We report on high prevalence of species *Enterovirus C, Enterovirus A and Enterovirus D* in Africa, Asia and Europe, respectively. Although this distribution broadly reflects the actual circulation of these viruses globally, the outcomes of this review are highly dependent on the (various) cohorts’ characteristics of the included studies.

The high prevalence of *Enterovirus D* in Europe may be partially explained by the high number of studies conducted in Europe that included respiratory samples specifically, as EV-D68 is known to cause predominantly respiratory disease. Several studies conducted on the African continent included asymptomatic participants (often children) of whom stool samples were obtained. This could have skewed the detections towards species *Enterovirus C,* as types within this species cause predominantly gastrointestinal symptoms, and the prevalence in children specifically is high. However, these findings are in line with findings in African environmental samples in which *Enterovirus C* strains are highly prevalent [49,50]. Similarly, in environmental samples from Europe, *Enterovirus B* strains are often predominant, in line with the results in this review [51,52,53,54]. *Enterovirus C* strains are also often found in environmental samples in Europe, suggesting a widespread circulation of this species in the European continent, which is in contrast with the low detection rate of *Enterovirus C* shown in this review.

Although showing a high prevalence of *Enterovirus A* in Asia, it was not the predominant species. This is in contrast with a previous review where *Enterovirus A* was the predominant species in this continent [3], and reports on outbreaks of HFMD and herpangina due to EV-A71, CVA6 and CVA16 [55,56]. As we excluded reports on type-specific disease (such as HFMD and herpangina), our results may more accurately reflect the overall circulation of the different EV types and species. However, as we excluded the type-specific disease in this review, we may underestimate the clinical relevance of these *Enterovirus A* types.

The information available on North and South America was limited. Though the USA has an EV surveillance system in place and frequently reports the results [2,14,57,58], only the most prevalent types are reported while a complete overview of all detected types is not provided. As a result, we excluded these reports in our current review. However, these reports describe a type distribution in line with the distribution in Europe; *Enterovirus B* appears the predominant species, followed by *Enterovirus D* and *Enterovirus A*. CVA21 was the only reported *Enterovirus C* type in these reports, while CVA9, CVB1-5, E6, E9, E11, E18, E25 and E30 were the most frequently reported types in *Enterovirus B*. EV-A71 and CVA16 were most frequently reported in *Enterovirus A* and EV-D68 was the only reported type in *Enterovirus D*.

An abundance of types was found to circulate globally. Type E30 showed an extraordinary peak in the proportion of genotypes, mainly in Europe, and was the most frequent EV detected in patients with neurological symptoms. This is in line with frequent reports of outbreaks of E30 related meningitis, both in Europe and in other continents [59,60,61,62], and with seroepidemiological studies reporting high rates of E30 seropositivity already in young age groups [63]. E30 has been reported to be genetically diverse, containing multiple lineages that co-circulate within populations [64,65]. The high prevalence of E30 may be the result of frequent emergence of lineages with a higher pathogenicity or transmissibility, or fluctuating immunity levels in the population [65,66].

EVs belonging to species *Enterovirus C* were rarely found in the studies conducted in Europe, while types belonging to this species appear to be abundant in Africa. While many EV types in *Enterovirus A* and *B* are detected in different sample types, most *Enterovirus C* types were found almost exclusively in stool samples, and *Enterovirus D* type EV-D68 was found almost exclusively in respiratory samples. Possibly, this indicates that the *Enterovirus C* and *D* types are limited to infecting a single organ system or cell type, while viruses belonging species *Enterovirus A* and *Enterovirus B* have a less exclusive cell tropism.

Surprisingly, the mean prevalence in asymptomatic subjects was higher than the mean prevalence in studies including specific patient groups. The EV infections detected in cohorts of healthy subjects may represent not only asymptomatic infections, but also reflect prolonged asymptomatic viral fecal or respiratory shedding after symptomatic infections. The high prevalence can further be explained by the fact that most studies on cohorts of asymptomatic subjects tested EVs in stool samples, which have a higher prevalence rate than other sample types. A total of twelve case-control studies were included in the current review. Ten of those case-control studies reported a lower EV prevalence in the control group than in the case group [25,67,68,69,70,71,72,73,74,75], while only two reported a higher EV prevalence in the control group [20,76]. This supports the hypothesis that the high median prevalence in asymptomatic participants is caused by limited comparability of the studies, rather than based on a real higher prevalence in asymptomatic individuals.

Although EVs are prevalent in children and adults, their prevalence in young children is higher as compared to adults.

Though much effort has been put into describing the epidemiology of EVs, the studies performed differ in many aspects, such as patient cohorts and collected sample types as highlighted in this review. The limited comparability between these studies, hampers the possibility to draw conclusions from aggregated information. To improve this, studies are needed that combine different clinical specimens or compare different patient groups. Specifically, asymptomatic and control matched cohorts are of interest. Most studies performed to date included patient groups with specified symptoms, though the majority of EV infections remains asymptomatic. Studies on asymptomatic participants or studies that include a control group will be important in estimating the real circulation of these viruses. By obtaining multiple sample types from these cohorts, the differences between the rates of and the EV types involved in respiratory and gastro-intestinal circulation can be further elucidated.

In this review, we chose to only include studies reporting on EV prevalence by PCR directed at the 5’UTR and studies reporting EV type distribution by typing based on VP1 sequences. Usage of these specific methods for studying EV epidemiology is advised by the European Non-Polio Enterovirus Network (ENPEN) [15], and exclusion of studies that do not meet these requirements has likely limited detection bias in prevalence values and underrepresentation of uncultivable EVs. However, this also vastly reduced the number of articles eligible for inclusion. We hope that with the ENPEN guidelines in place, studies that are to be conducted in the future will make use of the same, agreed upon methods, increasing comparability of studies, and allowing us to draw broader conclusions on overall EV epidemiology and disease burden.

## Figures and Tables

**Table 1:** Enterovirus types infecting humans by species [1]. CVA, Coxsackie A virus; CVB, Coxsackie B virus; EV, enterovirus; E, echovirus; PV, poliovirus.

Species	Types
*Enterovirus A* (*n* = 20)	CVA2–CVA8, CVA10, CVA12, CVA14, CVA16 EV-A71, EV-A76, EV-A89–EV-A91, EV-A114, EV-A119–EV-A121
*Enterovirus B* (*n* = 59)	CVA9 CVB1–CVB6 E1–E7, E9, E11–E21, E24–E27, E29–E33 EV-B69, EV-B73–EV-B75, EV-B77–EV-B88, EV-B93, EV-B97, EV-B98, EV-B100, EV-B101, EV-B106, EV-B107, EV-B111
*Enterovirus C* (*n* = 23)	CVA1, CVA11, CVA13, CVA17, CVA19–CVA22, CVA24 PV1–PV3 EV-C95, EV-C96, EV-C99, EV-C102, EV-C104, EV-C105, EV-C109, EV-C113, EV-C116–EV-C118
*Enterovirus D* (*n* = 4)	EV-D68, EV-D70, EV-D94, EV-D111

**Table 2:** Search as conducted in the PubMed Database on December 6th 2019. All identified publications were screened and in- or excluded following the inclusion and exclusion criteria.

**PubMed Database Search**
(((enterovirus[MeSH Terms]) OR enterovirus* [tiab]) AND (prevalence[MeSH Terms] OR epidemiology[MeSH Terms] OR prevalen* [tiab] OR epidemiolog* [tiab] OR “genotype”[MeSH Terms] OR genotyp* [tiab] OR serotyp* [tiab] OR type* [tiab] OR typing [tiab] OR (public health surveillance[MeSH Terms]) OR surveillan* [tiab])
**Embase/Ovid Database Search**
(exp Enterovirus/or enterovirus *.ti,ab,kw.) and (exp Prevalence/or exp Epidemiology/or exp Genotype/or exp Public Health Surveillance/or (prevalen * or epidemiolog * or genotype * or serotype * or type * or surveillan * or typing).ti,ab,kw.)
**Inclusion Criteria**
Observational studies reporting EV prevalence and/or type distribution found by detection or typing of EVs from samples that are routinely used for EV screening from a specified participant cohort.
**Exclusion Criteria**
Studies on comparison or validation of methods Studies on a single or a subset of EV types Studies on EV outbreaks Reporting in a cohort with type-specific disease (e.g., HFMD and myocarditis) Reporting in a cohort of participants with underlying diseases Articles not available in English No access to full text
**Exclusion Criteria for Reports on EV Prevalence**
Sample size < 100 Detection method other than PCR directed at the 5’UTR Studies describing prevalence of EVs and rhinoviruses (RVs) combined
**Exclusion Criteria for Reports on EV Type Distribution**
<10 samples reported Typing performed by any method other than by analysis of the (partial or full) VP1 sequence

**Table 3:** Frequency of quality scores for all 153 included papers. Quality was assessed using quality assessment forms (Tables S1 and S2).

**Quality Score**	2	3	4	5	6	7
**Number of Articles**	5	10	29	61	28	20

## Data Availability

No new data were created or analyzed in this study. Additional information on the analyses performed in this study will be provided on request.

## Supplementary Materials

The following are available online at https://www.mdpi.com/1999-4915/13/3/434/s1, Table S1: Quality Assessment Form Cross Sectional Studies, Table S2: Quality Assessment Form Case Control Studies, Table S3: List of all included studies.
