# Supplementary material for: World-Wide Prevalence and Genotype Distribution of Enteroviruses
Source: Viruses. 2021 Mar 8;13(3):434. doi: 10.3390/v13030434 (PMC7999254; doi:10.3390/v13030434)
Supplement: Supplementary file 1 [file viruses-13-00434-s001.pdf]

Article

World-Wide Prevalence and Genotype Distribution of Enteroviruses

Lieke Brouwer [1,2,*], Giulia Moreni [1,2], Katja C. Wolthers [1,#] and Dasja Pajkrt [2,#]

1 Department of Medical Microbiology, Amsterdam UMC, Location AMC
2 Department of Pediatric Infectious Diseases, Amsterdam UMC, Location AMC
# Authors contributed equally to this work
* Correspondence: l.brouwer@rotterdam.nl

Supplementary Material

Table 1. Quality Assessment Form Cross Sectional Studies.

Quality Indicator	Score
Study population is clearly specified (including symptoms)	1 pt
Subjects are selected from the same or similar populations	1 pt
Consecutive cases or random sampling	1 pt
Sample size is justified and satisfactory	1 pt
Response rate is satisfactory (>50%)	1 pt
The methods for detection and/or typing are sufficiently sensitive and specific	1 pt
Independent blind assessment of disease or record linkage	1 pt

Table 2. Quality Assessment Form Case Control Studies.

Quality Indicator	Score
Case definition is adequate with independent validation or record linkage	1 pt
Consecutive cases or random sampling	1 pt
Community controls	1 pt
Controls have no history of disease	1 pt
No significant difference in age between case and control group	1 pt
No significant difference in gender between case and control group	1 pt
The methods for detection and/or typing are sufficiently sensitive and specific, and the same in the case and control groups	1 pt

Table 3. List of all included studies.

Author	DOI	Included for Prevalence	Included for Type Distributio n	Contine nt	Collecti on date	Study duratio n	Samply type	Sympto ms	Age
Abedi et al.	10.1016/j.jcv.2019.04.005	YES	YES	4	4	1	2	4	1
Ahmad et al.	10.1002/jmv.23604	Yes	NO	2	5	3	4	2	1
Alcalá et al.	10.1186/s13099-018-0232-2	YES	NO	5	1	3	1	1	2
Andrés et al.	10.1016/j.jcv.2018.11.004	NO	YES	3	4	2	3	3	3
Ayukekbong et al.	10.1002/jmv.23926	YES	NO	1	3	1	1	4	4
Ayukekbong et al.	10.1016/j.jcv.2013.07.005	YES	NO	1	3	2	1	5	3
Benschop et al.	10.1128/JCM.01379-08	YES	NO	3	1	2	1	4	2
Benschop et al.	10.1016/j.diagmicrobio.2010.016	YES	NO	3	2	2	1	4	4
Biscaro et al	10.1016/j.meegid.2018.02.011	YES	NO	3	4	1	1	1	3
B'Krong et al.	10.1186/s12985-018-0980-0	NO	YES	2	2	5	5	4	4
Boaretti et al.	-	YES	NO	3	2	2	2	2	4
Bottcher et al.	10.1016/j.ijmm.2015.08.008	YES	YES	2	3	1	1	4	4
Bottcher et al.	10.2807/1560-7917.ES.2016.21.19.30227	YES	YES	3	3	2	3	3	4
Bozkurt et al.	-	YES	NO	2	3	2	1	1	3
Brittain-Long et al.	10.3109/00365548.2011.598876	YES	NO	3	2	2	3	4	4
Brouwer et al.	10.1007/s00705-018-3878-7	YES	YES	1	1	2	1	4	2
Bubba et al.	10.1097/INF.0000000000001344	YES	YES	3	3	3	5	4	2
Byington et al.	10.1542/peds.103.3.e27	YES	NO	4	1	1	5	4	1
Byington et al.	10.1542/peds.103.3.e27	YES	NO	4	1	1	5	4	1
Cabrerizo et al.	10.1002/jmv.24658	YES	NO	3	3	3	5	4	4
Cabrerizo et al.	10.1002/jmv.23693	YES	YES	3	2	1	2	2	4
Cabrerizo et al.	10.1002/jmv.24658	YES	NO	3	3	3	5	4	4
Chaimongkol et al.	-	YES	NO	2	2	1	1	1	2
Chakrabarti et al.	10.1007/s00431-018-3209-8	NO	YES	3	4	3	2	2	2
Chansaenroj et al.	10.1371/journal.pone.0182078	YES	NO	2	4	4	1	1	4
Chasqueira et a.	10.1016/j.ijid.2018.01.012	YES	NO	3	3	1	3	3	4

Chung et al.	10.1080/00365540600999126	YES	NO	2	1	2	3	3	3
Cordey et al.	10.1016/j.jcv.2015.05.003	YES	YES	3	3	1	4	4	4
Cordey et al.	10.1016/j.jcv.2017.01.008	NO	YES	3	4	2	5	2	3
Dalwai et al.	10.1186/1743-422X-7-236	YES	NO	2	1	3	2	2	3
de Crom et al.	10.1016/j.jcv.2016.01.014	NO	YES	3	3	3	5	4	3
de Jong et al.	10.1097/INF.0000000000001718	YES	YES	3	3	3	5	2	1
de Ory et al.	10.1002/jmv.23470	YES	NO	3	2	1	2	2	4
Di Cristanziano et al.	10.1016/j.jcv.2015.08.004	NO	YES	1	3	2	1	5	4
Do et al.	10.1371/journal.pone.0018176	YES	NO	2	2	3	3	3	3
Dumaidi et al.	10.1371/journal.pone.0172357	YES	YES	2	4	3	2	2	4
Dumaidi et al.	10.1371/journal.pone.0202243	YES	YES	2	4	1	2	2	4
Dupuis et al.	10.1002/jmv.22169	YES	NO	4	2	3	2	2	4
Etemadi et al.	10.1016/j.jviromet.2019.03.013	YES	NO	2	2	1	3	3	2
Faleye et al.	10.1155/2016/1412838	NO	YES	1	5	6	1	5	2
Farías et al.	10.1007/s00705-010-0828-4	YES	YES	5	2	2	2	2	4
Ferreira et al.	10.1371/journal.pone.0209993	YES	NO	5	3	1	2	2	4
Garcia et al.	10.1186/1743-422X-10-305	YES	NO	5	3	1	3	3	4
Gelaw et al.	10.1002/jmv.25765	YES	YES	1	4	1	1	1	2
Gosert et al.	10.1002/jmv.25005	YES	NO	3	4	2	1	1	4
Graf et al.	10.1186/s12879-019-4635-6	YES	YES	2	3	1	5	2	4
Gröndahl et al.	10.1007/s15010-013-0545-5	YES	NO	3	3	2	3	3	4
Guerra et al.	10.1002/jmv.24765	YES	YES	3	3	4	2	2	4
Han et al.	10.1016/j.jcv.2013.05.023	NO	YES	2	3	2	2	2	3
Hara et al.	10.1097/INF.0000000000000227	YES	NO	2	3	3	3	3	3
Harada et al.	10.1002/jmv.21454	YES	NO	2	2	4	1	1	4
Harvala et al.	10.1002/jmv.22023	YES	NO	3	2	3	2	2	4
Harvala et al.	10.1086/599094	YES	NO	3	2	2	2	2	4
Harvala et al.	10.2807/1560-7917.es2014.19.15.20772	YES	NO	3	3	2	2	4	4
Harvala et al.	10.1016/j.jinf.2014.02.017	YES	NO	3	3	1	4	2	1
Hassan et al.	10.1093/jpids/piy077	YES	NO	4	3	1	1	1	1

Hassan et al.	10.1093/jpids/piy077	YES	NO	4	3	1	1	5	1
Hasuwa et al.	10.1097/INF.0000000000002668	YES	NO	2	4	2	3	3	3
Hausfater et al.	10.1002/jmv.20068	YES	NO	3	1	2	2	2	3
Hellferscee et al.	10.1002/jmv.24869	YES	YES	1	3	3	3	3	4
Hellferscee et al.	10.1002/jmv.24869	YES	YES	1	3	3	3	5	4
Hellferscee et al.	10.1111/irv.12444	YES	NO	1	3	2	3	3	4
Hercik et al.	10.1371/journal.pone.0189712	YES	NO	1	4	2	3	4	4
Holtz et al.	10.1016.j.virol.2014.09.012	YES	NO	7	2	4	1	1	3
Hosoya et al.	10.1542/peds.107.1.e12	YES	NO	2	1	2	5	2	2
Jain et al.	10.4103/0972-9062.225835	YES	NO	2	4	3	2	2	4
Jain et al.	10.1007/s13337-016-0303-2	YES	NO	2	4	3	1	1	4
Jeong et al.	10.1007/s00705-010-0755-4	NO	YES	2	1	1	5	4	3
Karsch et al.	10.1097/INF.0000000000000802	YES	NO	3	3	2	1	2	3
Kim et al.	10.7883/yoken.JJID.2020.069	YES	NO	2	4	1	1	1	4
King et al.	10.1542/peds.2007-0252	YES	NO	4	1	4	2	2	1
Krasota et al.	10.3390/v8010010	NO	YES	3	3	2	2	2	4
Kumar et al.	10.1128/JCM.01483-12	YES	YES	2	2	2	2	2	3
Kumar et al.	10.3855/jidc.2413	YES	YES	2	2	2	5	4	3
Kumar et al.	10.3855/jidc.2413	YES	YES	2	2	2	5	5	3
Kumar et al.	10.1007/s00705-012-1476-7	NO	YES	2	2	1	2	2	3
Kumthip et al.	10.1016/j.meegid.2017.10.002	YES	YES	2	3	3	1	1	2
Lafolie et al.	10.1016/s1473-3099(18)30479-1	YES	NO	3	4	2	5	4	3
Le et al.	10.1371/journal.pone.0233117	YES	NO	2	4	2	3	3	4
Le et al.	10.1371/journal.pone.0233117	YES	NO	2	5	2	3	3	4
Lekana-Douki et al.	10.1186/1471-2334-14-373	YES	NO	1	3	2	3	3	4
Li et al	10.1016/j.jcv.2020.104516	YES	NO	2	4	2	2	2	3
Lu et al.	10.1016/j.jfma.2017.02.020	YES	NO	2	2	2	5	4	2
Lu et al.	10.1099/jmm.0.068247-0	YES	YES	2	3	3	3	3	3
Marinez planas et al.	10.1111/j.1469-0691.2011.03671.x	YES	NO	3	2	2	5	4	1

Markovich et al.	10.1097/INF.0000000000000627	YES	NO	2	3	2	3	3	3
Markovich et al.	10.1097/INF.0000000000000627	YES	NO	2	3	2	3	5	3
Mendoza et al.	10.1016/j.jinf.2006.11.013	YES	NO	5	1	1	2	2	4
Mitstchenko et al.	10.1016/j.jcv.2006.08.009	YES	NO	5	1	3	2	2	3
Moe et al.	10.1371/journal.pone.0159196	YES	NO	3	3	2	3	3	2
Molet et al.	10.1016/j.jcv.2015.11.024	NO	YES	3	3	1	5	4	4
Naga et al.	10.1556/030.2020.01059	YES	NO	1	6	6	3	3	3
Nahdi et al.	10.1002/jmv.23192	YES	NO	1	2	2	2	2	4
Naing et al.	10.1111/jpc.12113	YES	NO	6	2	2	1	4	1
Nairn et al.	PMID: 10447428	YES	NO	3	1	2	2	4	4
Nguyen et al.	10.1016/j.jcv.2016.07.014	YES	NO	2	3	3	3	3	4
Nguyen et al.	10.1002/jmv.25640	YES	NO	2	4	2	3	3	4
Nguyen et al.	10.1002/jmv.25640	YES	NO	2	4	2	3	5	4
Nokso-Koivisto et al.	10.1002/jmv.2161	YES	NO	3	1	1	3	4	3
O'Callaghan-Gordo et al.	10.1111/j.1365-3156.2011.02811.x	YES	NO	1	1	2	3	3	1
Okitsu et al.	10.1016/j.meegid.2019.104055	YES	NO	2	4	1	1	5	2
Osundare et al.	10.3390/v11111037	YES	YES	1	4	2	1	5	4
Othman et al.	10.1016/j.diagmicrobio.2015.10.019	YES	YES	1	3	2	2	2	4
Parisi et al.	10.1016/j.ejpn.2016.04.002	YES	NO	3	4	3	2	4	3
Patil et al.	10.1002/jmv.23992	YES	NO	2	2	3	1	1	2
Pellegrinelli et al.	10.1099/jgv.0.000937	YES	YES	3	4	4	3	3	2
Pellegrinelli et al.	10.1097/INF.0000000000002444	NO	YES	3	4	2	3	3	3
Pham et al.	10.1016/j.jviromet.2010.07.038	YES	NO	2	2	1	1	1	3
Pham et al.	10.1016/j.meegid.2018.03.009	YES	YES	2	4	2	1	1	3
Phan et al.	10.1002/jmv.20445	YES	NO	2	1	1	1	1	3
Piralla et al.	10.1016/j.diagmicrobio.2012.02.019	YES	NO	3	2	1	3	3	4
Pitkäranta et al.	10.1016/j.ijporl.2005.08.018	YES	NO	3	1	1	3	5	2
Pogka et al.	10.1016/j.jcv.2020.104349	YES	NO	3	4	2	5	4	4
Pogka et al.	10.1128/AEM.02872-16	YES	YES	3	3	4	1	5	3

Pretorius et al.	10.1093/infdis/jis538	YES	NO	1	2	2	3	3	4
Pretorius et al.	10.1016/j.jcv.2015.12.004	YES	NO	1	4	4	3	3	4
Pretorius et al.	10.1016/j.jcv.2015.12.004	YES	NO	1	4	2	3	4	4
Proenca-Modena et al.	10.1371/journal.pone.0042136	YES	NO	5	3	2	3	5	3
Puenpa et al.	10.1371/journal.pone.0098888	YES	YES	2	3	1	5	4	4
Rahimi et al.	10.5812/jjm.27113	YES	NO	2	3	2	2	2	2
Rathore et al.	10.1017/S0950268813003397	YES	NO	2	3	2	5	2	4
Ren et al.	10.1111/j.1469-0691.2009.02746.x	YES	NO	2	2	2	3	3	4
Renois et al.	10.1016/j.jinf.2013.03.007	YES	NO	3	2	1	3	3	3
Renois et al.	10.1016/j.jinf.2013.03.007	YES	NO	3	2	1	3	5	3
Rhedin et al.	10.1136/thoraxjnl-2015-206933	YES	NO	3	3	2	3	3	2
Rhedin et al.	10.1136/thoraxjnl-2015-206933	YES	NO	3	3	2	3	5	2
Richter et al.	10.1371/journal.pone.0220938	YES	YES	3	4	4	5	4	4
Richter et al.	10.1099/jmm.0.46447-0	NO	YES	3	1	2	5	2	4
Rihkanen et al.	10.1016/j.jpeds.2007.10.043	YES	NO	3	1	1	3	3	3
Rihkanen et al.	10.1016/j.jpeds.2007.10.043	YES	NO	3	1	1	3	3	3
Rimoldi et al.	10.1007/s00705-011-1037-5	YES	NO	3	2	2	1	1	3
Rocha et al.	10.1002/jmv.26216	YES	NO	5	4	1	2	2	4
Rotbart et al.	10.1097/00006454-199910000-00007	YES	NO	4	1	1	5	2	3
Rovida et al.	10.1016/j.diagmicrobio.2013.07.020	YES	NO	3	3	2	1	1	4
Saikruang et al.	10.1007/s00705-014-2191-3	YES	NO	2	2	1	1	1	4
Selim et al.	PMID: 18217322	YES	NO	1	1	2	5	2	4
shen et al.	10.1186/s12879-019-4162-5	YES	NO	2	4	3	2	2	3
shen et al.	10.1007/s00705-018-4021-5	YES	NO	2	4	1	1	1	3
shen et al.	10.1007/s00705-018-4021-5	YES	NO	2	4	1	1	5	3
Siafakas et al.	10.1016/j.mcp.2011.06.001	YES	YES	3	2	2	5	4	3
Silva et al.	10.1002/jmv.21231	YES	YES	1	1	1	1	1	3
Stellrecht et al.	10.1016/s1386-6532(02)00030-6	YES	NO	4	1	3	2	2	4
Subramoney et al.	10.1002/hsr2.59	YES	NO	1	3	2	3	3	4

Sung et al.	10.1002/jmv.21364	YES	NO	2	1	1	3	3	2
Tan Weng Jew et al.	PMID: 28064293	YES	NO	2	4	1	3	3	3
Tandukar et al.	10.3390/healthcare7010009	YES	NO	2	4	1	1	1	3
Tavakoli et al.	10.1016/j.jcv.2008.06.016	YES	YES	4	1	2	5	2	4
Thiberville et al.	10.1002/jmv.23265	YES	NO	3	2	1	3	3	4
Thiberville et al.	10.1186/1756-0500-7-81	YES	NO	3	3	1	3	4	4
Thongprachum et al.	10.1002/jmv.24155	YES	NO	2	3	3	1	1	3
Toh et al.	10.1093/ofid/ofz074	YES	NO	2	4	1	3	3	4
Tokarz et al.	10.1186/1743-422X-8-288	YES	NO	4	2	1	3	4	4
Tran et al.	10.5365/WPSAR.2012.3.2.001	YES	NO	2	2	2	3	3	4
Tryfonos et al.	10.1099/jmm.0.029892-0	YES	YES	3	2	3	5	4	4
Vidal et al.	10.1590/s0004-282x2011000400013	YES	NO	5	1	2	2	2	4
Vollbach et al.	10.1186/s12985-015-0427-9	YES	NO	3	2	5	2	2	3
Wei et al.	10.1097/MD.0000000000004870	NO	YES	2	4	1	2	2	3
Wieczorek et al.	PMID: 28520332	YES	NO	3	3	3	2	4	4
Wiertsema et al.	10.1002/jmv.22221	YES	NO	6	2	2	3	4	1
Wiertsema et al.	10.1002/jmv.22221	YES	NO	6	2	2	3	4	1
Wisdom et al.	10.1128/JCM.00993-09	YES	NO	3	2	1	3	4	4
Wu et al.	10.1371/journal.pone.0064889	NO	YES	2	3	1	1	5	2
Xiao et al.	10.1038/srep16639	YES	NO	2	3	2	3	3	3
Yew et al.	PMID: 28064293	YES	NO	2	4	1	3	3	3
Zhu et al.	10.1186/s40064-016-3194-1	NO	YES	2	2	2	5	2	3

DOI and first author are shown for each study. If DOI was not available, PMID is shown instead. Studies were included for analysis on prevalence, type distribution, or both. The data extracted from the studies are; Continent (1= Africa, 2 = Asia, 3= Europe, 4 = North-America, 5= South-America, 6 = Oceania, 7= studies conducted on samples from multiple continents), Date of last sample collection (1 ≤ 2006, 2 ≤ 2010, 3 ≤ 2014, 4 ≤ 2018, 5 ≤ 2020, 6 = Unknown), Study duration (1 ≤ 1 year, 2 = 1-3 years, 3 = 3-5 years, 4= 5-10 years, 5 > 10 years, 6 = Unknown), Sample type (1 = gastrointestinal, 2 = cerebrospinal fluid, 3 = respiratory, 4 = blood/serum/plasma, 5 = studies including multiple sample types), Symptoms (1= gastrointestinal, 2 = neurological, 3 = respiratory, 4 = studies including patient cohorts with other or multiple symptoms, 5 = no symptoms) and Age (1 ≤ 3 years, 2 ≤ 10 years, 3 ≤ 18 years, 4 > 18 years (the '>18' category includes studies in both children and adults in exclusively adults).
